# Cross-transmission in the Dental Office: Does This Make You Ill?

**DOI:** 10.1007/s40496-018-0201-3

**Published:** 2018-10-25

**Authors:** C. M. C. Volgenant, J. J. de Soet

**Affiliations:** 1 Department of Preventive Dentistry, Academic Centre for Dentistry of Amsterdam (ACTA), University of Amsterdam and Vrije Universiteit Amsterdam, Gustav Mahlerlaan 3004, 1081 LA Amsterdam, The Netherlands; 2 Department of Oral Kinesiology, Academic Centre for Dentistry of Amsterdam (ACTA), University of Amsterdam and Vrije Universiteit Amsterdam, Amsterdam, The Netherlands

**Keywords:** Infectious disease transmission, Dental infection control, Cross infection, Health care associated infection, Bacteria, Viruses

## Abstract

**Purpose of Review:**

Recently, numerous scientific publications were published which shed new light on the possible risks of infection for dental healthcare workers and their patients. This review aimed to provide the latest insights in the relative risks of transmission of (pathogenic) micro-organisms in the dental office.

**Recent Findings:**

Of all different routes of micro-organism transmission during or immediately after dental treatment (via direct contact/via blood-blood contact/via dental unit water and aerosols), evidence of transmission is available. However, the recent results put the risks in perspective; infections related to the dental office are most likely when infection control measures are not followed meticulously.

**Summary:**

The risk for transmission of pathogens in a dental office resulting in an infectious disease is still unknown; it seems to be limited in developed countries but it cannot be considered negligible. Therefore, maintaining high standards of infection preventive measures is of high importance for dental healthcare workers to avoid infectious diseases due to cross-contamination.

## Introduction

Most of the scientific publications in medicine investigate how to cure patients from diseases and this also applies for dentistry. Prevention of a disease is in most cases more cost effective than curing the disease [1]. For health care-associated infections, it has been described that the burden for the patient and his/her surroundings can also be substantial [2]. Prevention of disease becomes more and more important in an era where increased antibiotic resistance results in a rise in untreatable infections [3].

**Fig. 1:**
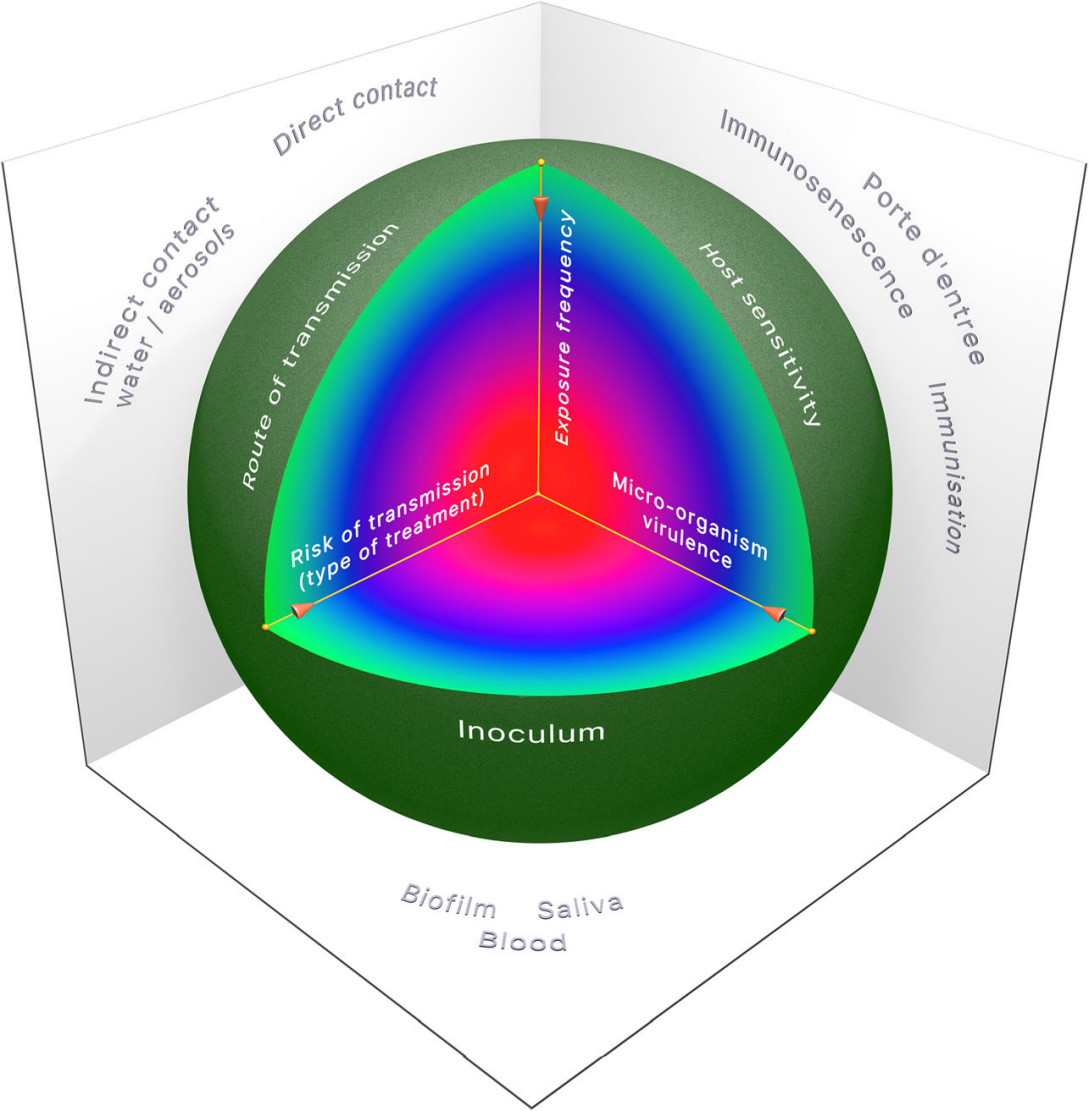
Visualisation of the three main factors responsible for infection risk, visualised on three axes. Note that the values of these axes are increasing to the centre. To obtain a relative infection risk, these three factors must be multiplied, resulting in either a more serious infection risk (red colour of the sphere), a moderate infection risk (blue colour) of negligible infection risk (green colour). On the edges of the sphere, the three main factors that determine whether transmission of a pathogen results in disease are shown. Outside the sphere examples are depicted of the involved factors in dentistry

Multiple factors are involved before transmission results in an infectious disease. The problem when studying cross-transmission is that it occurs everywhere, though transmission of pathogenic micro-organisms does not necessarily result in an infectious disease of the host. Figure 1 visualises the three main factors responsible for infection risk. Most important is the virulence of the micro-organism, or its pathogenicity class. It is unlikely that class 1 micro-organisms (which are not harmful for man, plant or animal) will cause an infectious disease. Likewise, a micro-organism will less likely cause an infection when not (frequently) in contact with a susceptible host or when the infectious dose of a micro-organism is not reached due to the type of treatment [4]. Therefore, the three factors risk of transmission (x-axis), micro-organisms virulence (y-axis) and exposure frequency (z-axis) should be multiplied in order to obtain a value that represents a relative infection risk [4]. The factors that are involved in this process can either increase or decrease the infection risk and are depicted in the sphere drawn in Fig. 1 with examples of these factors from dentistry presented around the sphere.

Both patients and dental health care professionals (DHCP) can serve as a host for micro-organisms; both patients and DHCP can serve as a reservoir for pathogenic micro-organisms and both can become infected because of their involvement in dental treatments. Transmission of micro-organisms in the dental office may occur by direct contact (including blood-blood contact) or by inhalation/ingestion of the micro-organisms (in (bio-)aerosols) from dental unit water. Recent reports on these different routes of transmission shed new light on the possible risks for DHCP as well as for patients receiving dental treatment. This review aims to give the latest insights in the relative risks of transmission of (pathogenic) micro-organisms in the dental office.

### Transmission by Direct Contact

Cross-transmission of micro-organisms in dental offices via direct contact is an almost unavoidable problem; it can occur via hands, improper sterilised instruments or needle stick accidents. The magnitude of this type of cross-transmission is difficult to estimate. Transmission of non-pathogenic micro-organisms will not result in medical problems since it will not result in an infectious disease (Fig. 1). Recent reports on cross-transmission in healthcare are therefore focusing on specific pathogenic micro-organisms that are easily identified and recognised because of their effect on the host.

One of these well-studied micro-organisms is MRSA, the methicillin-resistant *Staphylococcus aureus*, which is responsible for a substantial amount of health care-associated infections which are difficult to treat [5•]. Transmission of MRSA can occur by direct or indirect contact. It has been recently reported that the transmission dynamics of MRSA within a hospital environment are complex and that the likelihood of contamination with MRSA is possibly associated with the period of time patients stay in the healthcare facility [6]. In dentistry, where the contact time between patients and staff is relatively short, the transmission of MRSA is expected to be less complex.

MRSA is most frequently isolated in both the nose and the oral cavity [7–10]. Indeed, several studies in dentistry reported that MRSA is found more often in the nose or on hands of dental students compared to a control group without patient-contact, although contradicting findings have been reported [11–15]. The differences in prevalence may be due to differences between countries, e.g., in applying (hand)hygiene measures. These studies indicate that DHCP are more or less involved in MRSA transmission. The frequency of transmission via DHCP in the dental office is probably low and the main “porte de sortie” is most likely the patient. However, even if DHCP will become colonised by MRSA, the chances for further transmission are limited. For this to occur, the MRSA has to be transmitted to the next patient via air or direct contact. Transmission is possibly accompanied or amplified by infected surfaces, since surfaces have been reported to be frequently colonised by MRSA [13, 16–18]. Obviously, infection control measures will reduce the possibility for transmission of MRSA. Since (transmission of) MRSA is well-studied in health care, the results from the above named studies are assumed to be indicative for other micro-organisms such as *Escherichia coli*, *Klebsiella pneumoniae*, norovirus and *Candida albicans* for which transmission via direct contact is possible (Table 1).

**Table 1:** Some relevant infectious micro-organisms in a dental office sorted by their major transmission route

Transmission via direct contact	
Viruses	
Herpes simplex virus types 1 and 2	
Norovirus	
Coxsackievirus	
Bacteria	
*Staphylococcus aureus*	
*Escherichia coli*	
Transmission via blood-blood contact	
Viruses	
Hepatitis viruses (HBV, HCV, HDV*)*	
Human immunodeficiency virus (HIV)	
Bacteria	
*Neisseria gonorrhoeae*	
*Treponema pallidum*	
Transmission via dental unit water and aerosols	
Viruses	
Cytomegalovirus	
Measles virus	
Mumps virus	
Respiratory viruses (influenza, rhinovirus, adenovirus)	
Rubella virus	
Bacteria	
*Streptococcus pyogenes*	
*Mycobacterium tuberculosis*	
*Legionella pneumophila*	
*Pseudomonas aeruginosa*	

We have to realise that transmission is not synonym to infection. After transmission of a micro-organism to a host, the host will not become ill in most cases. Therefore, the colonisation of DHCP by MRSA is usually not noticed and will hence not result in disease or treatment. When conventional infection control measures are used (e.g. chemical or thermal disinfection, use of personal protective equipment), transmission of MRSA will be minimised. This cleaning and disinfection have to be performed with sufficient care since it has been reported that disinfection or removal of the biofilm is negatively affected when MRSA is present in a biofilm on (dry) surfaces [19–22]. Those dry surface biofilms can be transferred to the patient through the hands of the DHCP [23]. Hence, when the surrounding surfaces in a dental office are not sufficiently cleaned and disinfected, transfer of pathogens like MRSA is possible to occur in the dental office.

Another example of possible transmission via direct contact is the use of hollow instruments in dentistry. Several studies have been performed on the efficacy of the methods to clean and disinfect hollow instruments such as airotors and (high speed) handpieces, which is a recognised challenge in dentistry [24]. The presence of bacteria, fungi and viruses on and inside dental hollow instruments has been determined in a study by Andersen et al. [25]. Cleaning these handpieces using a tissue with ethanol (70%) is insufficient to eradicate microbial contamination [26]. In a commentary to this paper, it was stated that not only the exterior, but also the interior of these instruments should be cleaned and disinfected properly, since hollow instruments contain contamination of both the patient and the water/air supply [27]. Moreover, sufficient guidelines about how to decontaminate handpieces are available, but the majority of the DHCP is unaware of these guidelines [28].

The previously described studies indicate that the possibility for cross-transmission through dental equipment exists, although undeniable proof is difficult to obtain, probably due to incomplete reporting. One micro-organism of which transmission has been established in an oral surgery practice is hepatitis C virus (HCV). Molecular typing methods have been used to study the possible transmission of HCV in over 4000 patients after they visited a dental office [29, 30]. In total, 89 patients were found to be positive for HCV, but only two patients had identical strains of HCV which indicates transmission. The route of transmission in this case was the possible reuse of a contaminated vial during the application of intravenous drugs. Despite this mistake, the infection control measures in this particular oral surgery practice were inadequate [30, 31].

Another possible case of direct transmission via dental instruments was reported by newspapers from the UK, where a dentist did not clean the equipment properly. More than four and a half thousand patients were tested for HCV of which five patients were diagnosed with HCV [32]. No follow-up study has been published in scientific journals, so no clarity exists if these cases are connected.

These data suggest that transmission of pathogens between patients and dental equipment and vice versa does exist, in which the frequency of risk-contact will determine whether this contact will result in actual disease. But this will, as far as we know now, rarely cause infectious diseases.

### Transmission by Blood-Blood Contact

In the previous paragraph, it is discussed that the risks of insufficient cleaning and disinfection of instruments on the transmission of pathogens are not negligible. However, the highest risks of transmission in the dental office exist when pathogens are transported directly from blood (e.g., of the patient) to blood (e.g., of the DHCP). These blood exposure accidents (BEAs) are reported throughout the medical world, but especially within dentistry, there is a high risk for BEAs. DHCP frequently work with sharp instruments and needles while simultaneously they do not always have direct sight onto the working area or their own fingers. The risk of transmission of blood-borne pathogens is therefore a relevant occupational health risk [33].

A clear demonstration of this health risk is the elevated prevalence of seropositive hepatitis B individuals amongst DHCP. In the past, HBV infection was a relatively common occupational disease for DHCPs, but since vaccination against HBV has become obligatory in many countries for the dental profession, its prevalence dropped dramatically [34]. However, not all DHCPs are yet vaccinated against HBV and BEAs are worldwide responsible for 37–39% of the hepatitis B infections [35]. In an overview concerning transmission of blood-borne pathogens in the USA, Cleveland et al. found only three reports on cross-infection of HBV and HCV in the dental health care setting [36••]. Two reports dealt about an isolated transmission between patients and one study described transmission of HBV to three patients and two DHCPs. In all three cases, multiple deficiencies in infection control measures were observed while concurrently the occurrence of a BEA could not be excluded for these cases [30, 36••, 37, 38]. A review of studies which were performed in the Middle East and Northern Africa described that health care-related HCV transmission is responsible for over 50% of the HCV cases, although transmission in the dental surgery occurs in less than 5% of the reported cases [39].

After a BEA, active and appropriate post-accident prevention should be performed. Professional help is important to determine which measures have to be taken in order to prevent infection. It has been calculated that the actions after needle stick injuries are costly and cumbersome for the health care workers and the society [35]. A recent study from Pakistan suggested to develop educational programs and to install a health department managing BEAs to lower the threshold for DHCP of reporting accidents to fight present underreporting of BEAs [33]. In a study performed in The Netherlands, it was found that 16% of the BEAs reported to a professional counselling centre were considered high-risk incidents with a risk for the transmission of HBV as well as HCV and HIV, for what post-accident measures had to be taken [40]. In this study, it was reported that the vaccination rate for HBV of Dutch dentists was 98% and that 32% of the dentists who responded to the questionnaire reported to have at least 1 BEA in the previous year. This is a relatively high number compared to the medical environment and may be due to the frequent administration of local anaesthesia by dentists.

Medical and dental hospitals often have their own unit that manages BEAs. DHCP will only recognise the need for these units, when they have a sufficient level of knowledge about BEAs. Several studies, however, report that knowledge of both DHCP and students with respect to BEAs is insufficient [41–45]. Nevertheless, DHCP tend to estimate the risk level of a BEA themselves without professional counselling. It is therefore expected that the number of BEAs is much higher than the reported number and that the transmission of blood-borne pathogens between patient and DHCP will occur more frequently than reported. Since the number of infections in DHCP is not increased alarmingly, it is to be expected that the risk for infection is low. Consequently, the risk for a patient to be infected after a BEA is even much lower, especially when the DHCP is applying infection prevention measures in a correct way. Therefore, it is concluded that the risk for transmission of blood-borne pathogens in the dental office resulting in an infection is present, but acceptably low. Introduction of safety engineered devices or improved injection techniques to prevent BEAs have been recommended, but a clinical decrease in number of BEAs has not been reported yet [35, 46, 47].

### Transmission by Dental Unit Water and Aerosols

A third way of transmission of micro-organisms in the dental office is through the water of the dental unit water lines (DUWLs). The water from the DUWLs is used during treatments to cool the equipment, making this water essential for a safe dental treatment. Simultaneously, this cooling water is a possible source of (pathogenic) micro-organisms. Contamination of the water can occur from water backfiring from the patients’ side into the DUWLs as well as from the micro-organisms from the incoming water, with the latter being the main cause of contamination of the DUWLs [48].

Soon after the first use of the DUWLs [49], a multispecies biofilm is formed on the inside of the water lines. The moist environment of the DUWL, in combination with room temperature, the used fabrics of the DUWLs (polymers like polyurethane or polyvinyl chloride or silicone rubber tubing) and the relatively high surface for adherence form an ideal environment for biofilms to develop.

Both DHCP and patients are exposed to (contaminated) water from the DUWL directly (splatters/drinking) or indirectly (via aerosols through the air, produced by dental hand pieces) [50]. Aerosols are small liquid droplets (or solid particles) which float in the air. Aerosols and spatters can contain micro-organisms. These airborne micro-organisms can be spread in the dental office by ‘normal’ activities like talking, coughing and sneezing, but also by using dental instruments (e.g. airotors, ultrasonic instruments). Micro-organisms spread by aerosols can cause diseases like influenza, the common cold, but also respiratory diseases such as tuberculosis and legionnaire’s disease. Both the treatment room and the DHCP will become contaminated with micro-organisms from the DUWL [51–53]. Contamination due to aerosols or spatters is dependent of the correct application of the (high-volume) evacuator and the type of treatment [54].

When the DUWL water abundantly exceeds the microbiological quality requirements, this results in direct or indirect transmission of pathogens and may result in disease of susceptible hosts (Fig. 1). The most reported pathogens from contaminated water are *Legionella* species and *Pseudomonas* species, but also opportunistic genera such as *Propioniumbacterium*, *Mycobacterium* and *Stenotrophomonas* species are detected in the DUWL [55] (Table 1). *Pseudomonas aeruginosa* is frequently studied in waterlines because of its association with disease in susceptible hosts (i.e. cystic fibrosis). These pathogens are easily transmitted and originate from the main water tubing. Also, other species, such as *Achromobacter* species and mycobacteria, have been associated with infections from waterlines [49, 56–62]. Moreover, the presence of Gram-negative bacteria in the DUWLs can lead to the production of endotoxins (LPS) in the water and the air of a dental office [50, 63, 64].

*Legionella pneumophila* needs more attention since it is known to be able to cause Legionnaires’ disease, although it can also lead to Pontiac fever, an upper respiratory infection which often remains undiagnosed. Only inhalation of aerosols and choking or aspiration of *Legionella*-contaminated water can lead to infection. The presence of other bacteria in a biofilm does positively influence the survival of *L. pneumophila* [65]. But also the presence of amoeba is positively associated with *Legionella* because this bacterium can grow inside amoeba [66–69]. Many studies are focussing on the risks of having *L. pneumophila* in the water, but also non-*pneumophila* species of *Legionella*, like *Legionella anisa*, have been associated with infections [70, 71].

In dentistry, two cases of legionellosis have been reported recently [72, 73]. However, despite the fact that two people died from a *Legionella*-pneumonia after a dental treatment, it still is discussed whether (contaminated) DUWLs were the source of the *Legionella*, or that this bacterium had a different origin (Petti, 2016, Petti 2017a, Petti 2017b, Petti & Vitali, 2017). In either case, transmission of this bacterium did occur in the dental office.

When *Legionella* is present in DUWLs, it is to be expected that DHCP will develop antibodies against this bacterium in time. Several studies indicated that elevated levels of antibodies against *Legionella* occur in DHCP, but other studies contradict these results [74–76]. A meta-analysis by Petti and Vitali showed that the increased prevalence of anti-*Legionella* antibodies is highly dependent on the location of the study [77••]. Because there are too few studies where concentrations of *Legionella* in DUWL and antibodies against these bacteria have been studied simultaneously, this so called “chicken and egg dispute” cannot be solved yet [77••, 78–80]. Currently, no scientific evidence exists supporting an overall high occupational risk of *Legionella* infection. However, the above discussed studies together strongly indicate that transmission of pathogens from water to either patient or DHCP does occur, with a low risk for infection [81].

## Discussion

Cross-transmission of micro-organisms occurs frequently within the dental office. That is through direct and indirect contact between patients, DHCP and the outflow of DUWL. Based on the current research, this does not frequently result in infections in the patient or DHCP. Therefore, the actual risk for cross-infection is low, as far as we know from studies in developed countries. There is ample evidence that the same holds true for developing countries, where the hygiene level is much lower. Furthermore, with an ageing population in the developed countries, there will be more vulnerable patients in the dental office. Consequently, the likelihood that a cross-transmission will result in an infection will increase substantially.

Most studies describing cross-transmission in the dental office have been performed using bacteria as study target. It is suspected that DUWL contain many viruses (or phages). However, data on cross-infection from viruses such as measles virus are completely lacking, probably due to the limited available methods for molecular typing of viruses. It can be argued that transmission of viruses occurs with more ease and therefore more often compared to bacteria because of their smaller size. Due to the lack of studies on the relationship between cross-transmission and infection, especially focussing on viruses, the effect of this cross-transmission is not known.

Considering the research reports described in the current review, transmission resulting in infection cannot be excluded in the dental office. Consequently, maintaining a high standard of infection preventive measures must stay a main concern for DHCP, in order to keep themselves and their patients as healthy as possible. With this in mind, it is worrying that several studies conclude that the knowledge of DHCP about cross-transmission, cross-infection and how to prevent them is insufficient [41–45, 82, 83]. This should be kept in mind when planning post-graduate training programs and education programs of DHCP at all levels.

## Conclusion

The risk for transmission of pathogens in a dental office is still unknown but cannot be considered negligible. Usually, patients and DHCP do not develop infectious diseases after transmission. Due to increasing life expectancy of patients, improvement of health care causing more diseases to become chronic and the increasing virulence of micro-organisms (resistance), it is expected that in the future, transmission of pathogens results more frequently in development of infectious diseases. Therefore, infection control in the dental office should be considered as a mature and essential topic of which DHCP should be fully informed.
